# Editorial: Exploring the unique biodiversity of the Western Pacific to identify novel anti-infectious and anti-inflammatory compounds of natural origin

**DOI:** 10.3389/fphar.2023.1154627

**Published:** 2023-02-20

**Authors:** Philippe Georgel, Nicolas Lebouvier, Mariko Matsui

**Affiliations:** ^1^ Laboratoire d’ImmunoRhumatologie Moléculaire, Faculté de Médecine, Institut National de la Santé et de la Recherche Médicale (INSERM) UMR_S 1109, Institut thématique interdisciplinaire (ITI) de Médecine de Précision de Strasbourg, Transplantex NG, Fédération Hospitalo-Universitaire OMICARE, Fédération de Médecine Translationnelle de Strasbourg (FMTS), Université de Strasbourg, Strasbourg, France; ^2^ Institut des Sciences Exactes et Appliquées (ISEA) EA7484, Université de la Nouvelle Calédonie, Noumea, New Caledonia; ^3^ Group Bioactivities of NAtural compounds and derivatives (BIONA), Institut Pasteur of New Caledonia, Member of the Pasteur Network, Noumea, New Caledonia

**Keywords:** Western Pacific, biodiversity, bioactivity, phytochemistry, traditional medicine, natural products

Inflammation and many infectious diseases, such as sepsis, are still ongoing major health concerns. Although considerable therapeutic advances were made over the last decades, resulting in increased life expectancy, several hurdles remain to be addressed. Namely, antimicrobial resistance (AMR)—that led to more than 1.2 million deaths in 2019 (Murray et al., 2022)—or the adverse effects of several anti-inflammatory compounds are of utmost importance. Therefore, continuous efforts aiming at the discovery and development of novel molecules to deal with these challenges are required. For the past couple of years, research on natural products (NPs) used in traditional, herbal medicine, has been of high interest (Thomford et al., 2018) because such ancestral knowledge may reveal less toxic active compounds with fewer side effects.

The Western Pacific Region (WPR) comprises 37 countries and territories from China to New Zealand. Including 9 of the 36 established biodiversity hotspots (Kobayashi et al., 2019), the WPR therefore represents territories where medicinal plants belong to ecosystems threatened by human activities. Moreover, state members of the WPR show a strong acknowledgment of Traditional and Complementary Medicine (T&CM), with 93% of them reporting use of T&CM by their populations (WHO, 2019). Interaction between T&CM and conventional medicine, especially herb-drug interactions (HDI), can possibly cause ineffective treatment or adverse effects (Rosenkranz et al., 2012). Precise mechanisms of action and description of bioactive molecules participate in the prevention of HDI. In this Research Topic, bioactivities and phytochemistry of several traditional plants used in T&CM from the WPR countries are introduced, focusing on their chemical components and the related anti-infectious, immunoregulatory and healing properties.

Native from South-East Asia, *Calotropis gigantea* is an Apocyanaceae found in several Pacific Islands up to Australia and Central and South America. The whole plant is traditionally used to cure common skin diseases, including burns and small wounds. Roots and bark extracts are also used against leprosy, scabies and other infectious diseases. In their original research, Alafnan et al. studied the chemical composition and the biological properties of the *C. gigantea* leaves extracts. Phytochemical analyses showed that leave extracts contain 17 different compounds, mostly sesquiterpene, alkaloids and flavonoids. Ethanolic extracts of *C. gigantea* leaves exhibited considerable antioxidant activities. This paper also reports that plant extracts inhibited acetylcholinesterase (AChE) and tyrosinase enzymatic activities. *in vivo* upon topical application, these extracts improved wound healing following surgical- or burn-induced skin damage in Wistar rats.

*Helminthostachys zeylanica* is a fern-like plant belonging to the Ophioglossaceae family and native from the South-East Asia and Australia. This plant is traditionally used to treat diabetes, inflammatory and hepatic disorders and various infectious diseases. Shah et al. revealed that bacterial neuraminidase (BNA), which is an important player in several microbial infections and biofilm formation, is inhibited by the six known ugonin metabolites isolated from the rhizome of *H. zeylanica*. Interestingly, inhibitory activities of the compounds were higher than the reference (quercetin) and structurally related compounds (luteolin and eriodyctiol). Biofilm formation by *E coli* was also inhibited by the ugonin J in a dose-dependent manner, highlighting a potential antibacterial interest of the plant and these molecules.

Widespread in the South-West Pacific Islands, *Coleus forsteri* is an herbaceous Lamiaceae traditionally used to treat flu-like symptoms; it is also utilized as analgesic following ecchymosis. Only few studies characterized the chemical composition and biological activities of the plant. In this Research Topic, Nicolas et al. showed evidence of anti-inflammatory activities of *C. forsteri* whole extracts on human macrophages. Ethanolic and cyclohexane extracts of *C. forsteri* significantly decreased LPS-induced inflammatory cytokine IL-6 and TNF-α and quinolinic acid production. Seven known abietane diterpenes were characterized from *C. forsteri* cyclohexane extracts, including coleon U, horminone and family-related compounds which potentially contribute to the plant anti-inflammatory activities.

Among traditional Chinese medicine, Danggui Buxue Tang (DBT) is a formulae of two medicinal herbs containing root extracts of *Angelica sinensis* (Apiaceae) and *Astragalus membranaceus* (Fabaceae). DBT is recommended for patient recovery after a bone fracture, especially in aged women. Calycosin is the major active flavonoid found in *A. membranaceus* roots and in DBT. Using multi-omics approaches, the team of Kwan et al. showed that DBT and calycosin orchestrate osteoblastic differentiation and proliferation by regulating signaling pathways at RNA and protein levels, impacting on energy metabolism. Moreover, they showed that calycosin controls interaction with other DBT components during these processes.

In conclusion, this Research Topic reveals new biological properties and chemical characterization of four traditional plants and T&CM used within the WPR. It also highlights further explorations that are expected in this field to deepen our knowledge of their mechanisms of action.
